# Investigating the Mechanism of Trimethoprim-Induced Skin Rash and Liver Injury

**DOI:** 10.1093/toxsci/kfaa182

**Published:** 2021-01-04

**Authors:** Yanshan Cao, Ahsan Bairam, Alison Jee, Ming Liu, Jack Uetrecht

**Affiliations:** 1 Leslie Dan Faculty of Pharmacy, University of Toronto, Toronto M5S3M2, Canada; 2 Department of Pharmacology, College of Pharmacy and Pharmaceutical Sciences, University of Toledo Health Science Campus, Toledo, Ohio 43614; 3 Department of Pharmacology, University of Toronto, Toronto M5S3M2, Canada; 4 Leslie Dan Faculty of Pharmacy, Faculty of Medicine, University of Toronto, Toronto M5S3M2, Canada

**Keywords:** skin rash, idiosyncratic drug reaction, reactive metabolite, covalent binding, sulfotransferase

## Abstract

Trimethoprim (TMP)-induced skin rash and liver injury are likely to involve the formation of reactive metabolites. Analogous to nevirapine-induced skin rash, 1 possible reactive metabolite is the sulfate conjugate of α-hydroxyTMP, a metabolite of TMP. We synthesized this sulfate and found that it reacts with proteins *in vitro*. We produced a TMP-antiserum and found covalent binding of TMP in the liver of TMP-treated rats. However, we found that α-hydroxyTMP is not a substrate for human sulfotransferases, and we did not detect covalent binding in the skin of TMP-treated rats. Although less reactive than the sulfate, α-hydroxyTMP was found to covalently bind to liver and skin proteins *in vitro*. Even though there was covalent binding to liver proteins, TMP did not cause liver injury in rats or in our impaired immune tolerance mouse model that has been able to unmask the ability of other drugs to cause immune-mediated liver injury. This is likely because there was much less covalent binding of TMP in the livers of TMP-treated mice than TMP-treated rats. It is possible that some patients have a sulfotransferase that can produce the reactive benzylic sulfate; however, α-hydroxyTMP, itself, has sufficient reactivity to covalently bind to proteins in the skin and may be responsible for TMP-induced skin rash. Interspecies and interindividual differences in TMP metabolism may be 1 factor that determines the risk of TMP-induced skin rash. This study provides important data required to understand the mechanism of TMP-induced skin rash and drug-induced skin rash in general.

Trimethoprim (TMP) is an antibiotic that is commonly used in combination with sulfamethoxazole. This combination is associated with a wide variety of idiosyncratic drug reactions (IDRs) including skin reactions, hematological toxicities, liver injury, renal disorders, and other mild adverse reactions (Lawson and Paice, 1982). Skin rashes occur in 4%−24% of patients taking the combination of TMP and sulfamethoxazole (Brogden *et al.*, 1982; Iravani *et al.*, 1981), and the incidence of skin rash is 5 times higher in HIV-1 patients (Kovacs *et al.*, 1984). These IDRs are usually attributed to sulfamethoxazole, but TMP alone is associated with skin rashes, and rarely, liver injury (Kasanen and Sundquist, 1982; Koch *et al.*, 1973; Lindgren and Olsson, 1994; Singh and Singh, 1991). 

Most IDRs appear to be caused by reactive metabolites formed at the site of the adverse reaction rather than the parent drug. In previous studies with nevirapine, which causes an immune-mediated skin rash in female Brown Norway (BN) rats, our lab that a reactive sulfate metabolite formed in the skin was responsible for the rash. Specifically, topical application of a sulfotransferase (SULT) inhibitor prevented covalent binding and the skin rash where it was applied (Sharma *et al.*, 2013). The reactive sulfate metabolite was formed from 12-hydroxynevirapine, the major hepatic metabolite of nevirapine. Sulfotransferase (SULT) is one of the few drug-metabolizing enzymes in the skin with high activity. For example, minoxidil, used for hair growth, is metabolized to a sulfate in the skin, and it is the sulfate that is responsible for the pharmacological activity of the drug (Baker *et al.*, 1994). It is possible that there are other drugs that cause skin rashes by formation of a reactive sulfate in the skin similar to that of nevirapine. As with nevirapine, one of the metabolites of TMP is a benzylic alcohol which has the potential to be further metabolized to a reactive benzylic sulfate in the skin.

Although liver injury is less common that skin rash, TMP has also been reported to cause liver injury. It might be possible to model this with our impaired immune animal model. Two immune checkpoints in cancer therapy are the programed cell death 1 (PD-1) and cytotoxic T-lymphocyte-associated protein 4 (CTLA-4). Inhibiting these checkpoints may promote an immune response that can destroy the cancer cells. Similarly, inhibition of these checkpoints may lead to an animal model of idiosyncratic drug-induced liver injury (IDILI) similar to what occurs in humans. For example, when PD-1^−/−^ mice were cotreated with amodiaquine (a drug that causes IDILI in humans) and anti-CTLA-4, it resulted in elevations of ALT that remained elevated at least up to 8 weeks of treatment (Metushi *et al.*, 2015). Using this model with PD-1^−/−^ mice and anti-CTLA-4 treatment, the ability of other drugs including nevirapine and isoniazid, which cause IDILI in humans, was also unmasked (Mak and Uetrecht, 2015). It is possible that liver injury caused by TMP may also be unmasked.

We set out to test the hypothesis that TMP may form a reactive sulfate in the skin responsible for drug-induced skin rash by a mechanism analogous to nevirapine-induced skin rash. Female BN rats were used because the incidence of nevirapine-induced skin rash was 100%, whereas no male rats developed a rash (unless the dose of nevirapine was increased) (Shenton *et al.*, 2003). Mice were not used because they appear to lack the required SULTs to form the reactive benzylic sulfate of nevirapine that is responsible for the skin rash (Sharma *et al.*, 2013), and they might also fail to form a reactive sulfate of TMP. First, we produced a TMP antiserum, synthesized the reactive benzylic sulfate and tested its chemical reactivity, and finally tested the benzylic alcohol metabolite of TMP to see if it is a substrate for human SULTs (hSULTs). We also studied the covalent binding of TMP in the liver, which was previously shown to be caused by a reactive quinone methide formed by oxidation (Lai *et al.*, 1999) and investigated the potential of TMP-induced liver injury in our impaired immune tolerance mouse model.

## MATERIALS AND METHODS

###  

#### Chemicals and Reagents

Solvents (methanol, ethyl acetate, dichloromethane, acetonitrile, tetrahydrofuran, dimethylformamide) were obtained from Sigma-Aldrich (Oakville, Ontario) or Caledon (Georgetown, Ontario). The majority of chemical reagents, including manganese dioxide, sodium borohydride, phosphorous tribromide, sulfur-trioxide-triethylamine complex, triethylamine, pyridine, 4-bromobutyric acid, 1-ethyl-3-(-3-dimethylaminopropyl) carbodiimide hydrochloride, N′,N′-dicyclohexyl carbodiimide (DCC), N-hydroxysuccimide (NHS), and methylcellulose were obtained from Sigma-Aldrich unless otherwise noted. Silica (silica 60, 70 − 230 mesh) was obtained from Chromatographic Specialties (Brockville, Ontario). Trimethoprim was purchased from Alfa Aesar (Ward Hill, Massachusetts). POLYGRAM MN 300 CEL cellulose thin-layer chromatography (TLC) plates were products of Macherey-Nagel (Dueren, Germany).

#### Analytical Instruments

A Sciex API 3000 triple quadruple mass spectrometer was used for ESI-MS analysis with a Gemini 5 µ C18 110A 150 × 3.00 mm column (Phenomenex, Torrance, California). The mobile phase was a buffer containing acetonitrile: 0.1 mM ammonium formate, 1% formic acid (20:80) at a flow rate of 0.2 ml/min. Matrix-assisted laser desorption/ionization (MALDI) mass spectrometry was performed with a Bruker Autoflex MALDI-TOF to determine hapten density for drug-modified proteins. NMR spectra were obtained from 400 MHz Varian MercuryPlus NMR spectrometer. BCA assays, ALT assays, and other spectrophotometric assays were performed on the Biorad xMark microplate absorbance spectrophotometer. Cells stained for flow cytometry were analyzed on a BD LSR Fortessa cytometer.

##### Synthesis of trimethoprim-modified bovine serum albumin and ribonuclease conjugates

The TMP (5.6 g) and activated manganese (IV) oxide (17 g) were ground together until the 2 were evenly mixed. The solid mixture was stirred for 8 h at 120°C−125°C. The reaction mixture was cooled, then 150 ml of ethyl acetate:methanol (8:2, vol:vol) was added. The slurry was filtered and the yellow filtrate was concentrated, followed by separation on silica gel column chromatography using ethyl acetate:methanol (8:2, vol:vol) as the solvent. The TMP-ketone (TMP=O) ESI-MS fragments m/z: 305, 289, 275, 259, 244; RT 5.1 min and yield approximately 25%. TMP=O: ^1^H NMR (CD_3_OD, 400 MHz): 3.83 (s, 3H), 3.87 (s, 6H), 6.87 (s, 2H), and 8.25 (s, 1H).

Trimethoprim-ketone (TMP=O) (1.2 g) was dissolved in 50 ml of methanol, and 0.5 g of sodium borohydride was added to the solution. The mixture was refluxed for 5 h, then concentrated. Silica gel column chromatography using ethyl acetate:methanol (8:2, vol:vol) was used to obtain α-hydroxytrimethoprim (TMP-OH); yield 97%, ESI-MS fragments m/z: 307, 289, 274, 259, 243, 231 and RT 2.7 min. TMP-OH: ^1^H NMR (CD_3_OD, 400 MHz): 3.75 (s, 3H), 3.82 (s, 6H), 5.59 (s, 1H), 6.71 (s, 2H), and 7.44 (s, 1H).

The TMP-OH (0.01 g) was dissolved in 20 ml of anhydrous dichloromethane, followed by addition of 25 µl of phosphorus tribromide solution. The mixture was stirred for 5 h under nitrogen. Mercaptobutyric acid (0.015 g) was added to the reaction mixture. The reaction was stirred overnight under nitrogen, followed by evaporation of dichloromethane to give a slightly yellow liquid and a white precipitate along the side of the flask. The viscous liquid was dissolved in dichloromethane and extracted with water (pH 4). The organic layer was then evaporated. The TMP-thiobutyric acid adduct ESI-MS fragments m/z: 409, 289, 274, 259, 243; RT 2.7 min and yield 80%. Trimethoprim-thiobutyric acid adduct: ^1^H NMR (CD_3_OD, 400 MHz): 1.85 (m, 2H), 2.28 (t, 2H), 2.47 (t, 2H), 3.75 (s, 3H), 3.82 (s, 6H), 5.12 (s, 1H), 6.74 (s, 2H) and 7.65 (s, 1H).

The TMP-thiobutyric acid adduct (0.06 g) was dissolved in anhydrous dichloromethane. The NHS (0.024 g) was added followed by 0.019 g of DCC. The reaction was stirred overnight under nitrogen. The following day, the solution was washed using 0.1 M sodium bicarbonate solution followed by water. The organic layer was dried using anhydrous magnesium sulfate, filtered, and evaporated under a stream of nitrogen. The TMP-NHS ester ESI-MS fragments m/z: 506, 289, 259; RT 4.2 min and yield approximately 40%.

Bovine serum albumin (BSA) (Bioshop, Burlington, Ontario) (50 mg) was dissolved in 2 ml of 0.1 M potassium phosphate buffer pH 8. To the stirring solution of BSA, 40 mg of TMP-thiobutyric-NHS ester adduct dissolved in DMF was added dropwise to a final volume containing 15% DMF. The cloudy reaction was stirred overnight at 4°C followed by dialysis in water/PBS. Trimethoprim-modified-ribonuclease using ribonuclease purchased from Sigma-Aldrich was made using the same method as TMP-modified-BSA. Modified proteins were analyzed using MALDI. Mass spectrometry analysis showed 16 − 20 TMP-modified groups/BSA molecule.

##### Production of anti-TMP antiserum

Polyclonal antiserum was produced in female white New Zealand rabbits (Charles River, Montreal, Quebec) using a service provided by the University of Toronto in the Division of Comparative Medicine. Trimethoprim-modified-BSA was used as an immunogen to produce an anti-TMP-antiserum. Rabbits were immunized using 400 − 500 µg of TMP-modified-BSA protein combined with Freund’s complete adjuvant through subcutaneous injection. After 3 weeks, the animals were given a boost of 250 µg and subsequent boosts of 100 µg of TMP-modified-BSA (combined with Freund’s incomplete adjuvant). The animals were given 3 boosts of drug-modified proteins then sacrificed. Rabbits were anesthetized with isoflurane, followed by exsanguination by cardiac puncture. Two rabbits were immunized, and the serum from the rabbit with the better sensitivity was used for subsequent experiments.

##### Determination of the specificity of antisera using ELISA

Trimethoprim-modified-BSA conjugates (100 ng) were added to E105 microtiter plates (Cedarlane, Burlington, Ontario). Control wells were coated with 100 ng of BSA, TMP-modified-ribonuclease or ribonuclease. The plate was then incubated overnight at 4°C. The next day, the plate was washed, then blocked with 200 µl of 0.1% casein for 2 h on a plate shaker. Antiserum (1/25 000–1/625 000 dilution) was incubated on the blocked plate for 1 h at room temperature. The plate was washed, then incubated with 100 µl of goat-antirabbit IgG HRP (1/20 000) for 1 h. TMB reagent (100 µl, Bioshop) was added and incubated for 5 min in the dark. The reaction was then quenched using 100 µl of 1 N HCl. The absorbance was read at 450 nm.

#### Animal Care

Female BN rats were obtained from Charles River and housed 2 per cage with ad libitum access to water and powdered laboratory chow diet. Rats were obtained at 5 weeks of age, and at 9–10 weeks of age (approximately 150 g/weight), they were either maintained on a control chow (*n* = 3) or drug-containing diet (*n* = 4). Trimethoprim was mixed thoroughly with powdered chow for the 5-week study. Trimethoprim was provided at 0.2% in food for the first week, followed by 0.4% for 1 week, and 0.6% (wt:wt) thereafter. Serum was collected weekly and ALT was determined using an Infinity ALT kit (TR71121; ThermoScientific, Middleton, Virginia). Rats were scarified by CO_2_ asphyxiation followed by cervical dislocation. Liver, kidney, gut, spleen, and skin were collected.

Based on previous studies, we expected covalent binding to begin to level off by 3 days; therefore, for a 3-day study, rats were maintained with regular chow, and drug was administered by oral gavage daily. Rats were given 400, 400, and 100 mg/kg/day of TMP, TMP=O, or TMP-OH, respectively. Control rats were given 100 µl 0.5% methylcellulose in PBS by oral gavage. There were 3–4 rats in each group. After 3 days of treatment rats were scarified by CO_2_ asphyxiation followed by cervical dislocation. Liver, kidney, gut, spleen, and skin were collected.

Female PD-1^−/−^ mice (7–8 weeks) were bred in-house. Mice were separated into 2 groups: PD-1^−/−^ mice given regular rodent meal (*n* = 3 or 4) or PD-1^−/−^ mice given rodent meal with 2% TMP (wt/wt) (*n* = 4). Anti-CTLA-4 (clone 9D9; Bristol-Myers Squibb, Redwood City, California; 300 µg) was administered by i.p. injection weekly. Serum was collected weekly, and ALT was determined using an Infinity ALT kit (TR71121; ThermoScientific). Serum was also sent to The Centre for Phenogenomics (Toronto, Ontario) for GLDH analysis using a GLDH kit (Randox, Crumlin, United Kingdom). After 6 weeks of treatment, mice were sacrificed by CO_2_ asphyxiation, and their liver and inguinal lymph nodes were collected and processed immediately for flow cytometry analysis.

All animal protocols were approved by the University of Toronto Animal Care Committee and conducted in the Division of Comparative Medicine animal facility accredited by the Canadian Council on Animal Care. All procedures were in accordance with the Guide for the Humane Use and Care of Laboratory Animals.

##### Flow cytometry

At the end of treatment, mice were sacrificed, and livers were perfused with cold PBS to remove blood cells, then minced with scissors and subsequently digested for 30 min with digestion buffer (consisted with PBS, 0.05% collagenase, 1.25 mM CaCl2, 4 mM MgSO4, and 10 mM 4-(2-hydroxyethyl)-1-piperazineethanesulfonic acid (HEPES) at 37°C. After digestion, contents were passed through a 100 µm cell strainer, then resuspended with anticoagulant-citrate-dextrose (Acd) solution (consisting of PBS, 0.5% fetal bovine serum [FBS], 0.6% citrate-dextrose, and 10 mM HEPES). The liver cells were centrifuged at 30×g for 3 min, and the pellet containing hepatocytes was discarded. The supernatant was further centrifuged at 320×g for 5 min, and the pellet containing mainly nonparenchymal cells was resuspended in complete Roswell Park Memorial Institute media (RPMI media [Sigma-Aldrich], consisting of 10% FBS and 10 mM HEPES). The cells were then overlaid on 30% Percoll (GE Healthcare, Baie d’Urfe, Quebec) and centrifuged for 5 min at 800×g. The pellet was resuspended in Acd solution. An aliquot of each resuspended pellet was stained with trypan blue, and the concentration of cells was determined by cell counting using a hemocytometer. The cells (10^6^ cells, 100 µl/well) were plated in a V-bottom plate. After Fc receptors were blocked, cells were characterized on a BD LSR Fortessa cytometer, and the proportions of live lymphocytes were analyzed using FlowJo 10 software (Tree Star Inc, Ashland, Oregon). Lymphocytes were initially gated using forward-scatter (FSC) and side-scatter (SSC) and then gated on live cells. The following reagents were purchased from ThermoFisher Scientific: Antibodies against mouse CD3 conjugated to eFluor450 (cat no. 48-0032-82), CD4 conjugated to FITC (cat no. 11-0041-82), NK1.1 conjugated to PE-Cy7 (cat no. 25-5941), CD45R conjugated to Alexa700 (cat no. 56-0452-82), IL-17 conjugated to APC (cat no. 17-7277-81), and Foxp3 conjugated to PE (cat no. 12-4771-80); and fixable viability dye conjugated to eFluor506 (cat no. 65-0866-14). The antibody against CD8a conjugated to APC-Cy7 (cat no. 557654) was purchased from BD Biosciences.

In a separate group of mice, which had the same treatment as the above, the inguinal lymph nodes were taken upon sacrifice. The lymph nodes were passed through a 100 µm strainer, re-suspended in PBS, and centrifuged. The pellet was resuspended in 1% FBS in PBS. The remaining processing was the same as for liver to stain immune cells.

##### Separation of epidermal and dermal skin layers

Skin from the back of each rat was removed and placed on dry ice upon sacrifice. Skin sections were stretched on a Petri dish and digested with 0.25% trypsin-EDTA solution (Life Technologies, Burlington, Ontario) at 4°C. The skin sections tend to curl; therefore, they were stretched using forceps during the digestion and allowed to float overnight at 4°C. Trypsin solution was then replaced with fresh solution and the skin floated for another 6–8 h at 4°C. Epidermal skin was peeled off the dermal layer, washed with distilled deionized water and PBS 3 times, then homogenized in 1:10 of cell lysis buffer (Cell Signaling Technologies, Pickering, Ontario): Protease inhibitor (Halt Protease Inhibitor 100 X EDTA-free, Pierce, Rockford, Illinois). The dermal layer was removed from the remaining hypodermis and fat, washed with distilled deionized water and PBS 3 times, then homogenized in cell lysis buffer with protease inhibitor (1:10). Skin samples (350–400 mg) were homogenized using a Polytron 2100 homogenizer using 750 µl of cell lysis buffer with protease inhibitor. Homogenized samples were centrifuged at 16 100×g for 10 min, the fat layer and debris were discarded, whereas the clear supernatant was stored at −80°C. Whole skin sections, cleaned of hair, hypodermis, and fat, were prepared the same way except without digestion with 0.25% trypsin-EDTA solution. Skin homogenates were evaluated for covalent binding of TMP.

##### Preparation of rat S9 liver fractions and other tissue homogenates

Liver, kidney, gut (flushed with 10 ml of PBS), and spleen from rats were homogenized in cell lysis buffer with protease inhibitor (1:10). Homogenates were centrifuged at 9000×g for 20 min. The fat layer and the debris were discarded, and the supernatant was stored at −80°C. Tissue homogenates were studied for covalent binding of TMP.

##### Synthesis of the sulfate conjugate of TMP-OH (TMP-S)

**Scheme 1.**
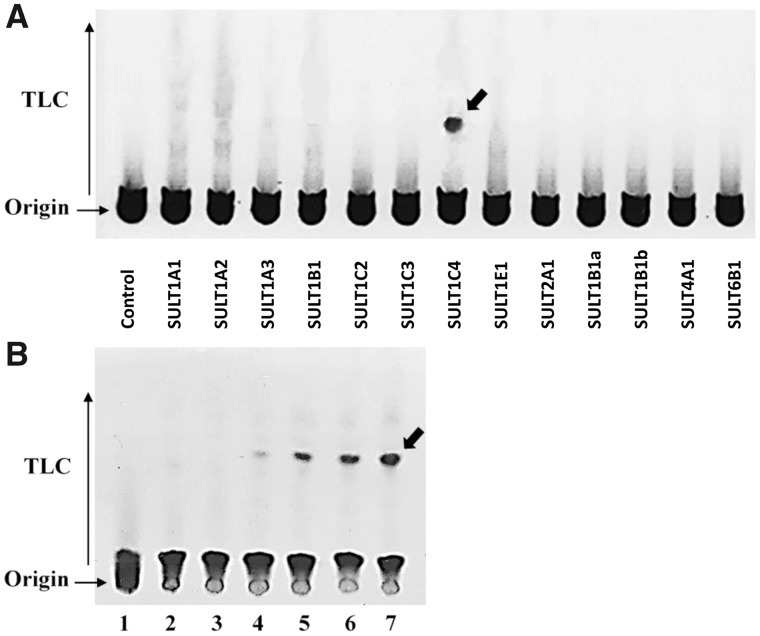
Schematic of (A) synthesis of protein conjugates and (B) synthesis of TMP-S. MeOH, methanol, DCM, dichloromethane; DMF, dimethylformamide; THF, tetrahydrofuran, PBS, phosphate-buffered saline.

Oven dried TMP-OH (0.006 g) was dissolved in 6 ml of anhydrous tetrahydrofuran containing 10 µl of triethylamine and 4 A molecular sieves. Sulfur trioxide-triethylamine complex (0.01 g) was added to the stirring solution. The reaction was stirred overnight at 4°C. The sulfate was purified using silica chromatography (chloroform:methanol [85:15, vol:vol]) (Scheme 1). TMP-S ESI-MS fragments m/z: 385, 305, 388; RT 8.1 min; yield 40%.

##### Reactivity of TMP and its metabolites with liver or epidermal skin homogenates

Liver and epidermal skin homogenates were freshly prepared from female Sprague Dawley rats (Charles River) 8–10 weeks of age to study *in vitro* covalent binding. To investigate the reactivity of the TMP-OH and TMP-S with liver and skin proteins, 2.5 mg of TMP-OH or TMP-S was dissolved in 100 µl of DMF (an equivalent of 8.2 mM or 6.5 mM final concentration, respectively) and added to a stirring solution of 1 mg/ml of liver S9 homogenate or epidermal skin homogenate in PBS (1 ml volume). The mixture was stirred overnight at 4°C and covalent binding of TMP-OH and TMP-S were detected using immunoblotting assay.

To compare the reactivity of TMP, TMP=O and TMP-OH covalent binding to liver S9 proteins, 1 mg of each metabolite was dissolved in 100 µl of DMF (equivalent of 3.4, 3.3, and 3.2 mM concentration, respectively) and added to freshly prepared liver S9 proteins. The reaction was stirred overnight at 4°C.

To compare the binding of TMP-OH to liver and skin proteins over time, 1 mg of freshly prepared liver or skin proteins was incubated with 5 mM of TMP-OH for up to 1 week. TMP-OH may be further bioactivated by liver enzymes to a reactive metabolite that contributes to its covalent binding. Inhibition of various metabolic pathways was studied. Freshly prepared liver S9 fractions (1 mg) were preincubated with 5 mM of 1-aminobenzotriazole (Sigma-Aldrich), 4-nitrophenol (Sigma-Aldrich) and 1-phenyl-1-hexanol (Tokyo Chemical Industry, Toshima, Japan) or (-)-borneol (Alfa Aesar, Tewksbury, Massachusetts) for 30 min at room temperature, followed by addition of 5 mM of TMP-OH to each reaction mixture and incubated overnight. The reaction mixtures were immunoblotted to study covalent binding of TMP-OH.

##### Covalent binding using SDS-PAGE and immunoblotting

All protein concentrations were determined using the BCA assay (Pierce). Protein samples (20 and 40 µg for S9 liver and epidermal skin proteins, respectively unless otherwise stated in the figure legend) were combined with SDS sample buffer and heated for 5 min at 95°C. For testing the specificity of the polyclonal TMP-antiserum, antiserum diluted to 1:5000 was preincubated with TMP at a final concentration of 40 µg/ml for 2 h prior to use in the immunoblotting assay.

Proteins were separated using freshly made polyacrylamide gels (4% stacking and 8% resolving gel) by first stacking for 35 min at 80 V, followed by resolving at 125 V using the Bio-Rad mini-gel system. Bio-Rad Precision Plus Protein Kaleidoscope Prestained Protein Ladder was used as the reference. After separation, proteins were transferred at 25 W for 1 h, 45 min onto a 0.45 µm nitrocellulose membrane (Biorad) using the Bio-Rad Mini-Transfer system, followed by blocking for 1.5 h at room temperature with 5% skim milk (Bioshop). Polyclonal antiserum was incubated overnight at 4°C. The next day, blots were incubated with 1:20 000 goat-antirabbit IgG HRP detection antibody (Sigma-Aldrich, St Louis, Missouri) for 1.5 h, followed by washing to remove detection antibody. The western blots were incubated for 5 min in Clarity Western ECL Substrate stain (Bio-rad, Mississauga, Ontario) and imaged using ChemiDoc imager (Bio-rad). Glyceraldehyde 3-phosphate dehydrogenase (GAPDH) loading control was probed for all membranes. Membranes were stripped using Restore Western Blot Stripping Buffer (Pierce) for 12 min at room temperature, followed by blocking with 5% skim milk for 1 h and then incubated in 1:5000 dilution of mouse monoclonal HRP-anti-GAPDH (Pierce) for 1 h. The blots were stained and imaged as described above.

##### Preparation of human cytosolic SULTs and sulfotransferase assay

Recombinant hSULTs (SULT1A1, SULT1A2, SULT1A3, SULT1B1, SULT1C2 SULT1C3, SULT1C4, SULT1E1, SULT2A1, SULT2B1a, SULT2B1b, SULT4A1, and SULT6B1), expressed using pGEX-2TK or pET23c prokaryotic expression system, were prepared as described previously (Pai *et al.*, 2002; Sakakibara *et al.*, 2002; Suiko *et al.*, 2000). The sulfating activity of purified recombinant hSULTs toward TMP-OH was assayed based on a previously established procedure (Bairam *et al.*, 2018), with radioactive PAP[^35^S] as the sulfate donor. The assay mixture, following a 15 min reaction at 37°C, was separated by TLC and analyzed for [^35^S]sulfated TMP-OH. The solvent system used was n-butanol/isopropanol/88% formic acid/water (3:1:1:1; by volume). Upon completion of TLC, autoradiography was performed to reveal the [^35^S]sulfated TMP-OH present in the assay mixture.

## RESULTS

###  

#### Characterization of Anti-TMP-Antiserum

**Figure 1.**
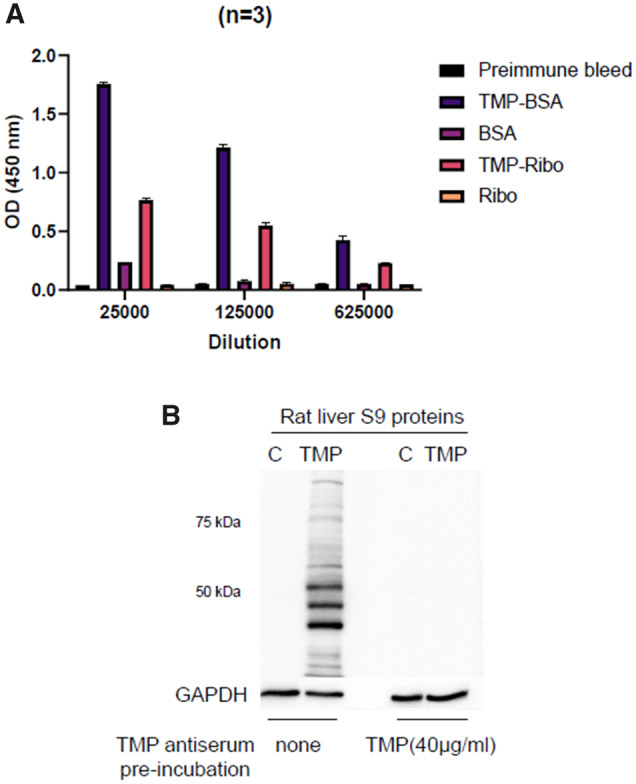
ELISA analysis and immunoblot analysis of antiserum specificity. A, ELISA analysis of TMP-antiserum and preimmune serum binding to TMP-BSA conjugate (TMP-BSA), TMP-ribonuclease conjugate (TMP-Ribo), and ribonuclease (Ribo) at different dilutions of serum. B, Immunoblot analysis of BN rat liver S9 proteins from TMP-treated rats (0.2%−0.6% in food) for 5 weeks (TMP) and regular diet (C) using TMP-antiserum with or without preincubation with 40 µg/ml with TMP. Protein loading was 20 µg of protein per lane, and TMP-antiserum was diluted 1:5000. Error bars represents mean ± SEM of 3 experiments.

Anti-TMP serum specificity was determined using an ELISA assay and immunoblot to determine covalent binding to endogenous TMP-modified proteins from rats treated with TMP. The rabbits’ antisera with a higher titer as determined by ELISA assay was used for subsequent immunoblotting. Figure 1A shows the ELISA responses to various protein conjugates. Trimethoprim antiserum binding to the TMP-BSA conjugate signal was inhibited in the presence of TMP-ribonuclease, and the TMP-antiserum did not bind to ribonuclease (Figure 1A). Liver proteins from TMP-treated BN rats were western blotted as a positive control to test the ability of the antiserum to detect endogenous covalent binding. Covalent binding to a wide range of liver proteins was detected, and preincubation of the antiserum with 40 µg/ml of TMP blocked the observed covalent binding (Figure 1B).

#### Detection of *In Vivo* Covalent Binding of TMP to Rat Liver and Skin Proteins

**Figure 2.**
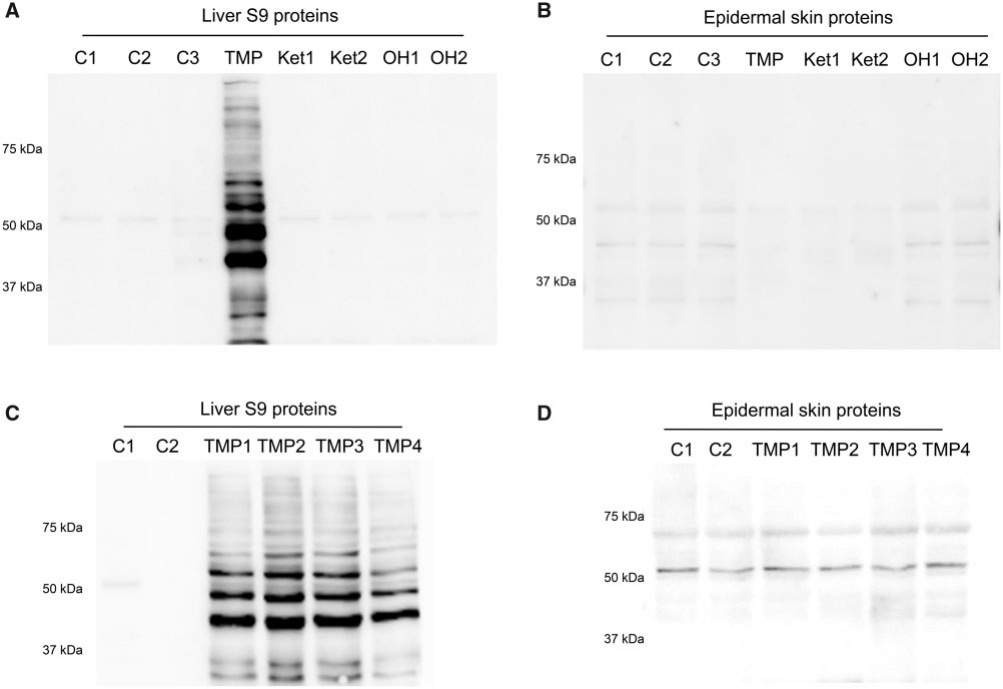
Immunoblot analysis of (A) BN rat liver S9 proteins and (B) epidermal skin proteins after 3 days of treatment with 400 mg/kg/day of TMP, 400 mg/kg/day of TMP=O (Ket1, Ket2), 100 mg/kg/day of TMP-OH (OH1, OH2) or 100 µl of 0.5% methylcellulose in PBS by gavage (C1−C3); (C) BN rat liver S9 proteins and (D) epidermal skin proteins from treatment with 0.2%–0.6% TMP in food for 5 weeks (TMP1−TMP4) or regular rodent meal (C1−C2). Numbers 1 − 4 denotes 4 different rats. Protein loading was 20 µg of liver protein and 40 µg of epidermal skin protein; primary antibody was diluted 1:5000 and 1:2000, respectively.

Covalent binding of TMP to a wide range of proteins was detected in the liver (Figure 2). For BN rats treated with TMP (400 mg/kg/day) for 3 days, covalent binding was detected in the liver but not in the epidermal skin (Figs. 2A and 2B). Brown Norway rats treated with TMP=O (400 mg/kg/day) or TMP-OH (100 mg/kg/day) for 3 days did not show covalent binding in the liver or in the epidermal skin (Figs. 2A and 2B). In addition, there was also no covalent binding detected in whole skin, dermis layer, spleen, kidney, or gut proteins for 3 days treated rats (data not shown). At 5 weeks of TMP treatment, covalent binding was detected in liver proteins but not epidermal skin protein (Figs. 2C and 2D). Even though there was covalent binding in the liver, there was no significant difference in ALT between control and TMP-treated rats (data not shown) after 5 weeks of treatment.

#### Reactivity of TMP-S and Covalent Binding of TMP and Its Metabolites to Liver and Epidermal Skin Homogenates *In Vitro*

**Figure 3.**
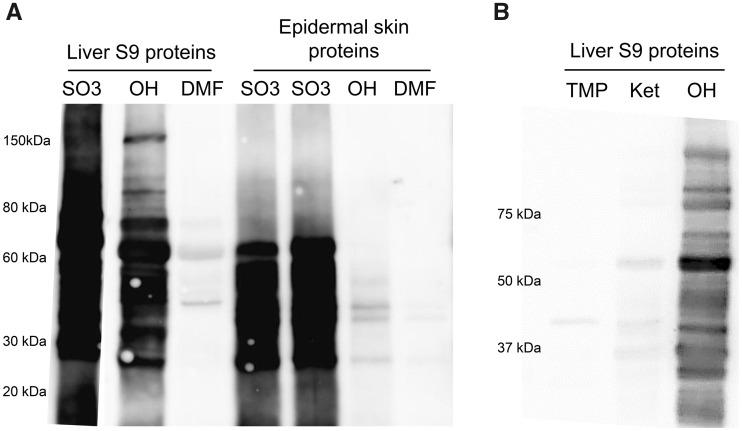
*In vitro* covalent binding of TMP and its metabolites to liver and epidermal skin proteins isolated from SD rats. Immunoblot analysis of (A) S9 liver or epidermal skin proteins incubated with TMP-S (SO_3_) or TMP-OH (OH), with a DMF solvent control; B, S9 liver proteins incubated with TMP, TMP=O, or TMP-OH. Protein loading was 25 µg, and the primary antibody was diluted 1:1000.

Trimethoprim-S was synthesized to test the ability of the sulfate to covalently bind to proteins. TMP-S is reactive and covalently binds to a wide range of S9 liver and epidermal skin proteins (Figure 3A). Trimethoprim-OH was also found to covalently bind to proteins, although the binding to hepatic proteins was much greater than that to skin proteins (Figure 3).

#### TMP-OH is Sufficiently Reactive to Covalently Bind to Proteins

**Figure 4.**
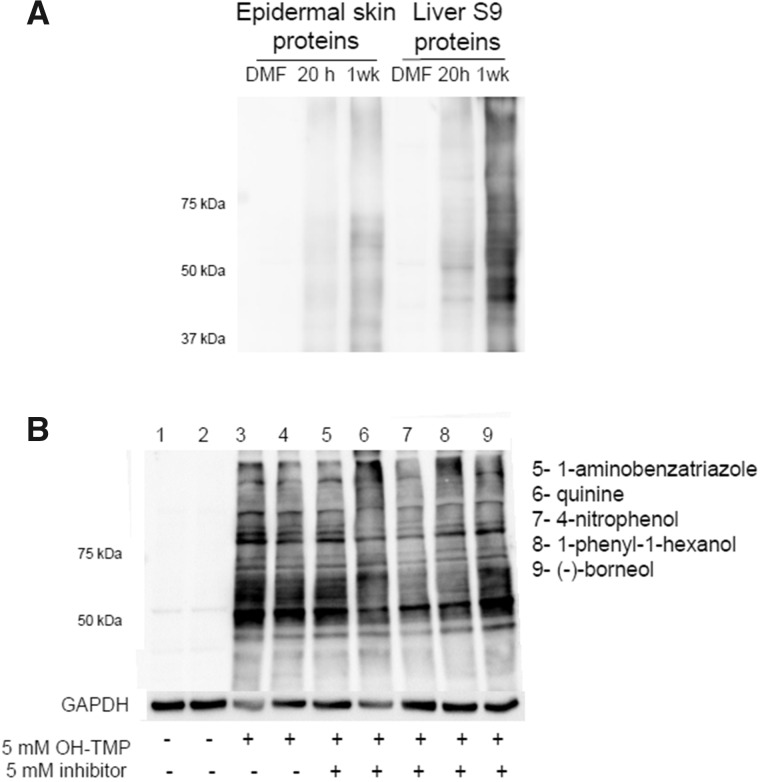
Immunoblot analysis of (A) epidermal skin or liver proteins incubated with 5 mM TMP-OH for 20 h or 1 week; B, freshly prepared S9 liver proteins incubated with various inhibitors (5 mM) for 30 min followed by incubation with 5 mM of TMP-OH for 20 h. Fifty micrograms of protein were loaded, and the primary antibody was diluted 1:1000.

Further investigation demonstrated that TMP-OH covalently binds to proteins, and binding increased with time (Figure 4A). There was less covalent binding to epidermal skin proteins than to liver proteins (Figure 4A). Because liver proteins were freshly isolated prior to incubation with TMP-OH, it could be possible that metabolic enzymes in the liver may further bioactivate TMP-OH to a reactive metabolite contributing to the covalent binding observed. The chemical structure of TMP-OH makes it a potential target for further oxidation, sulfation, and glucuronidation. The addition of 1-aminobenzantriaole for nonselective inhibition of cytochromes P450s, quinine for inhibition of CYP3A and CYP2D1, 4-nitrophenol and 1-phenyl-1-hexanol for inhibition of SULTs, and (-)-borneol for inhibition of UDP-glucuronosyltransferases did not decrease the amount of covalent binding of TMP-OH (Figure 4B).

#### The Potential of TMP to Cause Liver Injury in PD-1^−/−^ Mice Cotreated With Anti-CTLA-4 and TMP

**Figure 5.**
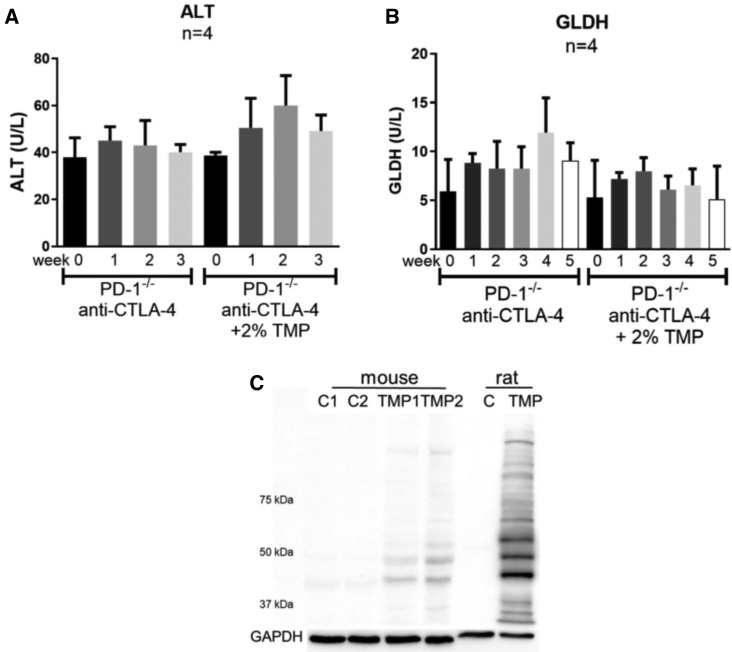
Trimethoprim did not cause liver injury in female PD-1^−/−^ mice co-treated with anti-CTLA-4 and 2% TMP in food. A, Serum ALT up to 3 weeks; B, serum GLDH up to 5 weeks. C, Immunoblot analysis of liver proteins from TMP-treated mice and BN rat (5 weeks). Protein loading was 20 μg, and primary antibody was diluted 1:5000. Values represent the mean ± SD. Analyzed for statistical significance by the unpaired t test ANOVA. p<0.05 was considered significant.

We investigated the potential of TMP to cause liver injury using in the PD-1^−/−^ mice co-treated with anti-CTLA-4. No increase in ALT or GLDH was observed when the PD-1^−/−^ mice co-treated with anti-CTLA-4 were treated with TMP (Figs. 5A and 5B). However, there was a decrease in Th17 cells in the liver and a decrease in T cells in the lymph nodes of TMP-treated mice (Supplementary Figs. S4 and S5). Covalent binding of TMP in the liver of mice was detected, but it was much less compared with the rats treated with TMP (Figure 5C).

#### Analysis of the TMP-OH-Sulfating Activity of hSULTs

**Figure 6.**
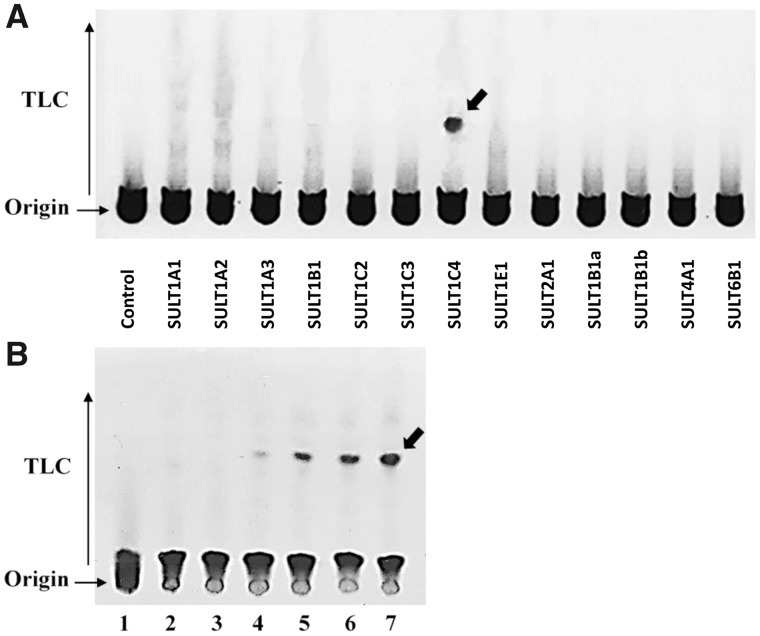
Analysis of the TMP-OH-sulfating activity of human SULTs. A, the results from a systematic analysis of all thirteen known human SULTs. B, the results from a concentration-dependent experiment using SULT1C4. The figure shows the autoradiographs taken from the TLC plates used for the analyses. In (A), lanes correspond to a control without enzyme and assay mixtures catalyzed by SULT1A1, SULT1A2, SULT1A3, SULT1B1, SULT1C2, SULT1C3, SULT1C4, SULT1E1, SULT2A1, SULT2B1a, SULT2B1b, SULT4A1, and SULT6B1, respectively. The final concentration of the substrate (TMP-OH) tested was 250 µM. The arrow indicates the position of [^35^S]sulfated TMP-OH. In (B), Lanes 1–3 correspond to control with substrate (TMP-OH; dissolved in DMSO) but without enzyme (SULT1C4), control without substrate and DMSO but with enzyme, and control without substrate but with DMSO and enzyme, respectively. Lanes 4 − 7 correspond to assay samples containing 50, 250, 500, and 1000 µM of TMP-OH, respectively.

A systematic analysis of the TMP-OH -sulfating activity of hSULTs was first performed. Of the 13 hSULTs, only SULT1C4 displayed significant sulfating activity toward TMP-OH (Figure 6A). The specific activity determined for SULT1C4, at 250 µM substrate (TMP-OH) concentration, was 0.943 nmol/min/mg. A concentration-dependent experiment was subsequently carried out. As shown in Figure 6B, the amount of [^35^S] sulfated TMP-OH produced increased proportionately with increasing concentrations of TMP-OH tested as substrate.

## DISCUSSION

The mechanisms of IDRs are not well understood. Most IDRs, especially skin rashes, appear to be immune mediated, and most appear to be caused by reactive metabolites rather than the parent drug. Most reactive metabolites are formed in the liver but are too reactive to reach the skin in significant concentrations. However, SULTs are present in the skin and have the potential to convert some drugs or metabolites to chemically reactive species. As mentioned, we had previously shown that the skin rash caused by nevirapine is due to a reactive benzylic sulfate formed in the skin. Another drug that causes skin rash and has the potential to form a reactive benzylic sulfate is TMP. Therefore, we set out to test the possibility that the benzylic alcohol metabolite of TMP (TMP-OH) can form a sulfate metabolite in the skin leading to covalent binding in the same species and strain used for the nevirapine experiments. Detecting covalent binding in the epidermis of rats is more challenging than in the liver because it is only 2–3 cells thick, and we did not observe significant covalent binding of TMP in the skin of TMP-treated rats. In contrast, there was a large amount of covalent binding in the liver. It was previously shown that TMP can be readily oxidized by hepatic microsomes to a reactive iminoquinone methide that reacts with N-acetylcysteine (Goldman *et al.*, 2016; Lai *et al.*, 1999). However, this metabolite is not likely to reach the skin or be formed in the skin in significant amounts. Even though there was a large amount of covalent binding in the liver, it is not surprising that we did not see liver injury because the dominant immune response in the liver is immune tolerance. We thought it might be possible to produce an animal model of TMP-induced liver injury in our impaired immune tolerance model in mice. However, we found that there was far less covalent binding of TMP in mice than in rats, and this is a plausible explanation of why we did not see an immune response with liver injury in this mouse model.

We found that the benzylic sulfate is chemically reactive; however, TMP-OH is not a substrate for the major hSULTs, which makes the benzylic sulfate a less attractive candidate for the cause of TMP-induced skin rash. We were surprised that TMP-OH was chemically reactive without further metabolism, although it was less reactive than the sulfate. This observation had also been made recently by others (Nolte *et al.*, 2020). This may be because the amino groups on the heterocyclic ring can help to stabilize an intermediate in the reaction of TMP-OH with nucleophiles. There was also less covalent binding of TMP-OH to skin proteins than to hepatic proteins. The major protein in the epidermal skin is keratin, whereas in the liver a wide range of proteins are present. Keratin is a protein with low cysteine content (Fuchs, 1983), and TMP-OH is most likely to react with thiol nucleophiles. However, even a small amount of covalent binding to a specific minor protein in the skin could lead to an immune response because the skin is much less tolerogenic than the liver. It is possible that the TMP-OH is metabolized to a glucuronide, which would likely be more reactive than the alcohol; however, inhibition of glucuronidation by borneol did not decrease the covalent binding to hepatic proteins *in vitro*.

Given our data, we suggest that covalent binding of TMP-OH to proteins in the skin is most likely responsible for TMP-induced skin rash; however, we cannot exclude the possibility that a minor SULT is present in some patients that can bioactivate TMP-OH. It is, of course, possible that the skin rash is caused by the parent drug by a mechanism that does not involve covalent binding of the drug to proteins or some other pathway such as O-dealkylation of 2 of the methoxy groups to form a catechol, which could be further oxidized to an ortho-quinone. Although there is little cytochromes P450 in the skin, oxidation to an ortho-quinone could be carried out by a peroxidase. Except for the studies with hSULTs, our studies were performed in rats and mice, and interspecies and interindividual differences in the metabolism of TMP could be partially responsible for the idiosyncratic nature of TMP-induced skin rash.

## SUPPLEMENTARY DATA

Supplementary data are available at Toxicological Sciences online.

## DECLARATION OF CONFLICTING INTERESTS

The authors declared no potential conflicts of interest with respect to the research, authorship, and/or publication of this article. 

## FUNDING

This work was funded by grants from the Canadian Institutes for Health Research (93647, 142329). 

## Supplementary Material

kfaa182_Supplementary_Data
