# Supplementary material for: Investigating the Mechanism of Trimethoprim-Induced Skin Rash and Liver Injury
Source: Toxicol Sci. 2021 Jan 4;180(1):17–25. doi: 10.1093/toxsci/kfaa182 (PMC7916736; doi:10.1093/toxsci/kfaa182)
Supplement: kfaa182_Supplementary_Data [file kfaa182_supplementary_data.docx]

**Supplementary Material**

Investigating the Mechanism of Trimethoprim-Induced Skin Rash and Liver Injury

Yanshan Cao, Ahsan Bairam, Alison Jee, Ming Liu, and Jack Uetrecht

Yanshan Cao, Leslie Dan Faculty of Pharmacy, University of Toronto, Toronto Canada, M5S3M2

Ahsan Bairam, Department of Pharmacology, College of Pharmacy and Pharmaceutical Sciences, University of Toledo Health Science Campus, Toledo, OH 43614, USA

Alison Jee, Department of Pharmacology, University of Toronto, Toronto Canada, M5S3M2

Ming Liu, Department of Pharmacology, College of Pharmacy and Pharmaceutical Sciences, University of Toledo Health Science Campus, Toledo, OH 43614, USA

Jack Uetrecht, Leslie Dan Faculty of Pharmacy and Faculty of Medicine, University of Toronto, Toronto Canada, M5S3M2

Corresponding Author: Jack Uetrecht, Faculty of Pharmacy, University of Toronto, 144 College Street, Toronto, Ontario, Canada M5S 3M2.

Table S1: Serum concentration of TMP, TMP=O and TMP-OH measured using LC/MS/MS of drug treated animals. nd = not detectable. Lowest standard concentration was 0.4ug/ml for TMP and 0.04ug/ml for TMP=O and TMP-OH.

| **Animal, Treatment and timepoint** | **TMP (ug/ml)** | **TMP=O (ug/ml)** | **TMP-OH (ug/ml)** |
| --- | --- | --- | --- |
| Mice TMP (400 mg/kg/day), 2 hrs | 12.7 | <0.04 | 1.4 |
| Mice TMP (400 mg/kg/day), 6hrs | 1.4 | <0.04 | <0.04 |
| Mice TMP (400 mg/kg/day), 24 hrs | nd | nd | nd |
|  |  |  |  |
| BN rat TMP (400 mg/kg/day), 2 hrs | 3.2 | <0.04 | 0.5 |
| BN rat TMP (400 mg/kg/day), 6 hrs | 2.9 | nd | 0.4 |
| BN rat TMP (400 mg/kg/day), 22 hrs | <0.4 | nd | <0.04 |
|  |  |  |  |
| BN rat TMP=O (400 mg/kg/day), 2 hrs | nd | 1.0 | <0.04 |
| BN rat TMP=O (400 mg/kg/day), 6 hrs | nd | 0.4 | nd |
| BN rat TMP=O (400 mg/kg/day), 22 hrs | nd | nd | nd |
|  |  |  |  |
| BN rat TMP-OH (100 mg/kg/day), 2 hrs | nd | 0.04 | 1.7 |
| BN rat TMP-OH (100 mg/kg/day), 6 hrs | nd | <0.04 | 1.1 |
| BN rat TMP-OH (100 mg/kg/day), 22 hrs | nd | nd | 0.4 |


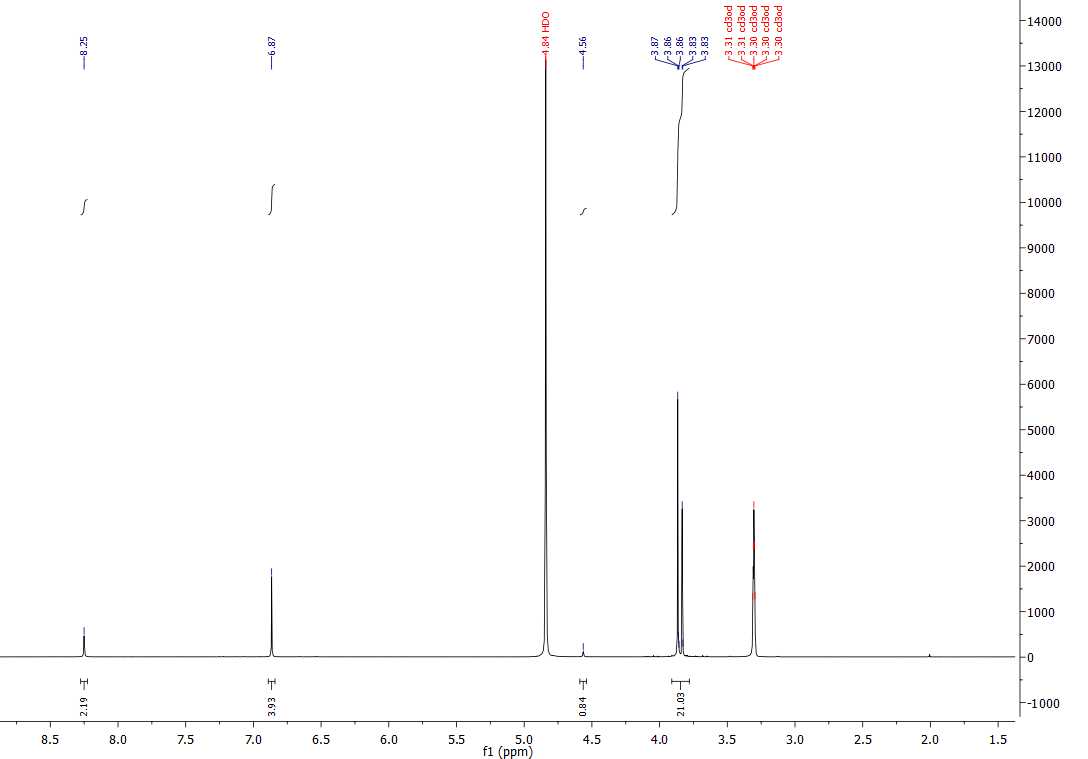


Figure S1: TMP=O ^1^H NMR (CD_3_OD, 400 MHz): 3.83 (s, 3H), 3.87 (s, 6H), 6.87 (s, 2H) and 8.25 (s, 1H)


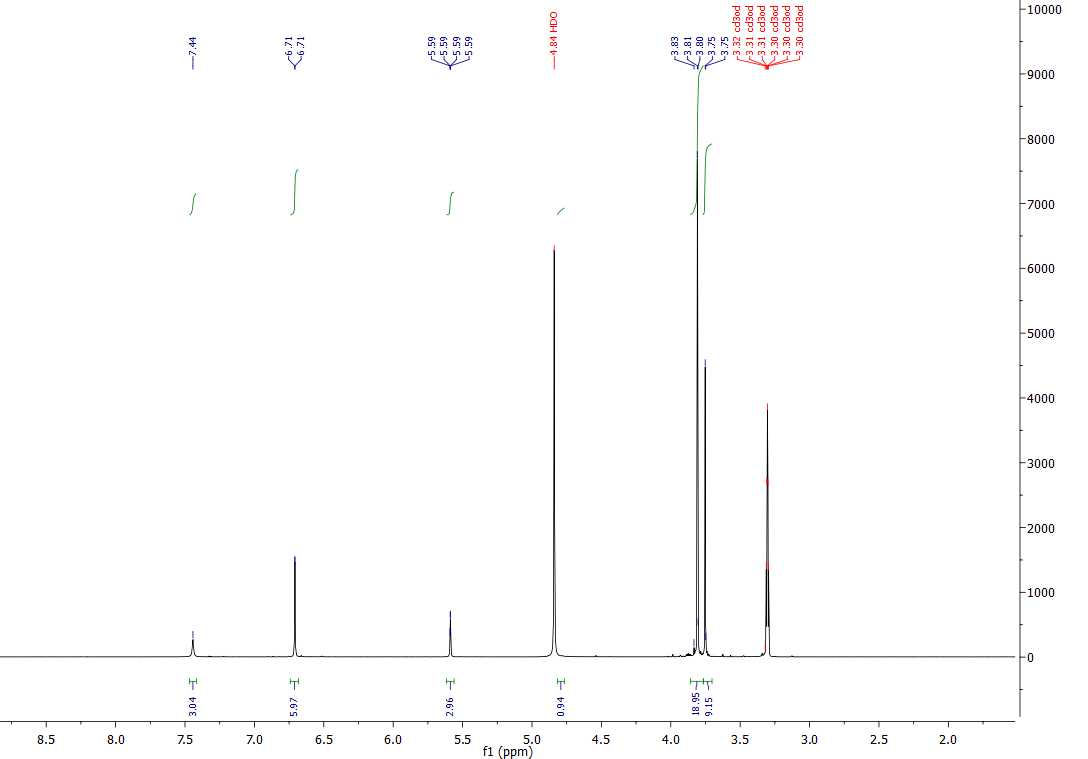


Figure S2: TMP-OH ^1^H NMR (CD_3_OD, 400 MHz): 3.75 (s, 3H), 3.82 (s, 6H), 5.59 (s, 1H), 6.71 (s, 2H) and 7.44 (s, 1H).

Figure S3: TMP-thiobutyric acid adduct ^1^H NMR (CD_3_OD, 400 MHz): 1.85 (m, 2H), 2.28 (t, 2H), 2.47 (t, 2H), 3.75 (s, 3H), 3.82 (s, 6H), 5.12 (s, 1H), 6,74 (s, 2H) and 7.65 (s, 1H).


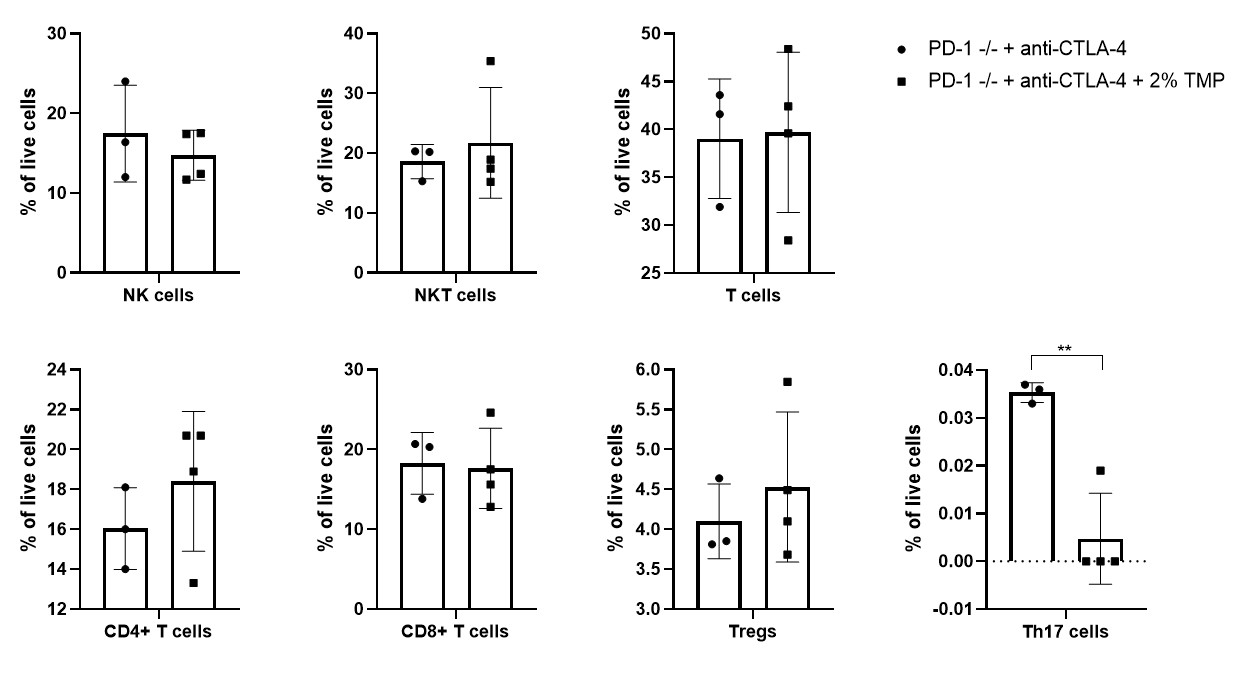


Figure S4: Flow cytometry analysis of mononuclear leukocyte in liver of female PD-1^-/-^ mice after 6 weeks treatments of anti-CTLA4 antibody alone (PD-1 -/- + anti-CTLA-4) and anti-CTLA4 antibody + 2% TMP in food w/w (PD-1 -/- + anti-CTLA-4 + 2% TMP). Values represent the mean ± SE. Analyzed for statistical significance by unpaired t test ANOVA. P<0.05 was considered significant.


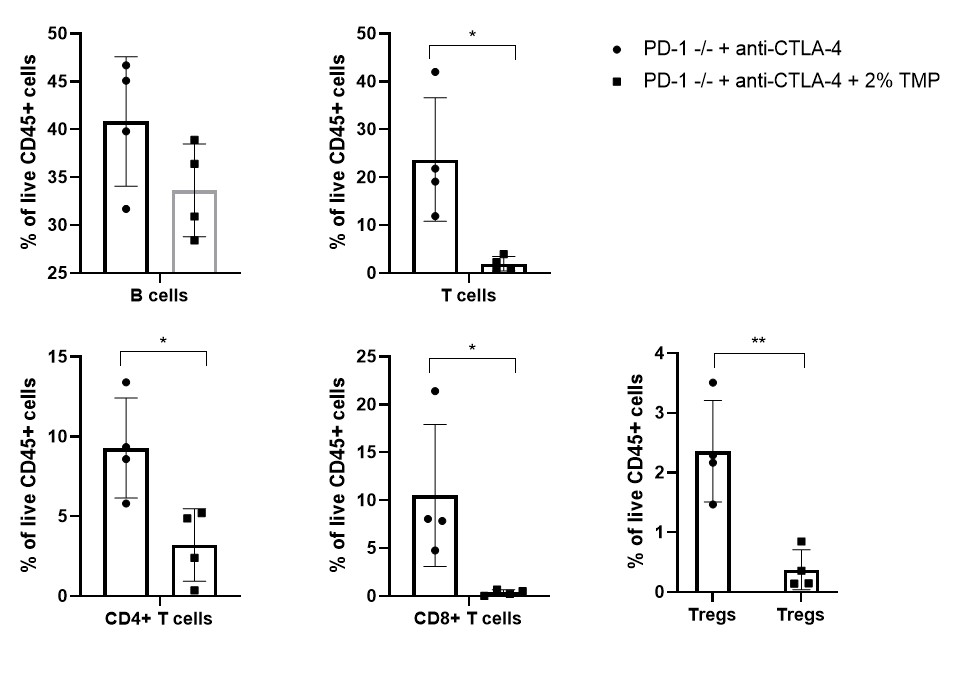


Figure S5: Flow cytometry analysis of mononuclear leukocyte in inguinal lymph nodes of female PD-1^-/-^ mice after 6 weeks treatments of anti-CTLA-4 antibody alone (PD-1 -/- + anti-CTLA-4) and anti-CTLA4 antibody + 2% TMP (in food w/w) (PD-1 -/- + anti-CTLA-4 + 2% TMP). Values represent the mean ± SE. Analyzed for statistical significance by unpaired t test ANOVA. P<0.05 was considered significant.
